# Supplementary material for: MYH9 Facilitates Cell Invasion and Radioresistance in Head and Neck Cancer via Modulation of Cellular ROS Levels by Activating the MAPK-Nrf2-GCLC Pathway
Source: Cells. 2022 Sep 13;11(18):2855. doi: 10.3390/cells11182855 (PMC9497050; doi:10.3390/cells11182855)
Supplement: Supplementary file 1 [file cells-11-02855-s001.zip › cells-1845724-supplementary.pdf]

Table S1. List of the primers used in the present study.

Gene	Primer Sequences (5' -> 3')
MYH9	F: GAGCAAATGGGCCTGCT
	R: TGTTGTCGGGCATGGA
GCLC	F: AACTCAGACATTGGATGGAG
	R: ACCTTTGACAGTGGAATGAG
GCLM	F: AGGGAGTTTCCAGATGTCTT
	R: AAGCAATGATCACAGAATCC
GPX2	F: TCATTGCCAAGTCCTTCTAT
	R: TGTTCAGGATCTCCTCATTC
GSS	F: TCCATGTGATCCGACGAACA
	R: CACCTTCTTAGTCCCAGCCA
MnSOD	F: CAAAGGGGAGTTGCTGGAAG
	R: AGCAGTGGAATAAGGCCTGT
Catalase	F: GAGCCTACGTCCTGAGTCTC
	R: CCGGATGCCATAGTCAGGAT

Table S2. List of the primary antibodies used in the present study.

Antibody	Catalog number (Company)
MYH9	ab138498 (abcam, Cambridge, UK)
GCLC	ab53179 (abcam, Cambridge, UK)
GLCM	GTX114075 (GeneTex, Irvine, CA, USA)
GPX2	GTX100292 (GeneTex, Irvine, CA, USA)
p-GSK3b	#9336 (Cell Signaling Technology Danvers, MA, USA)
p-PDK1	#3061(Cell Signaling Technology Danvers, MA, USA)
p-Akt(S473)	#4060 (Cell Signaling Technology Danvers, MA, USA)
p-Akt(T308)	#9275 (Cell Signaling Technology Danvers, MA, USA)
Akt	#9272 (Cell Signaling Technology Danvers, MA, USA)
p-Erk	#4370 (Cell Signaling Technology Danvers, MA, USA)
p-p38	#9215 (Cell Signaling Technology Danvers, MA, USA)
p-JNK	#4668 (Cell Signaling Technology Danvers, MA, USA)
Erk	#9102 (Cell Signaling Technology Danvers, MA, USA)
p38	#9212 (Cell Signaling Technology Danvers, MA, USA)
JNK	#9252 (Cell Signaling Technology Danvers, MA, USA)
GAPDH	MAB374 (EMD Millipore, Billerica, MA, USA)

Table S3. Patient characteristics and clinical information of the TCGA-HNSC dataset.

Characteristic	No. (%) of HNC patients
**AGE**	38-85
Mean age±SD (years)	60.87±11.87
**AGE SPECIFIC GROUP**	
<40	18 (3.5%)
40-59	64 (12.3%)
50-59	151 (29.0%)
60-69	174 (33.5%)
≥70	112 (21.5%)
Not Available	1 (0.2%)
**GENDER**	
Male	384 (73.8%)
Female	136 (26.2%)
**RACE**	
White	445 (85.6%)
Black or African American	47 (9.0%)
Asian	11 (2.1%)
American Indian or Alaska native	2 (0.4%)
Not Available	15 (2.9%)
**HPV STATUS**	
Positive	21 (4.0%)
Negative	65 (12.5%)
Not Available	434 (83.5%)
**PRIMARY SITE**	
Tongue	156 (30.0%)
Larynx & Oropharynx & Hypopharynx	136 (26.2%)
Oral Cavity	73 (14.0%)
Floor of mouth	62 (11.9%)
Others	93 (17.9%)
**CLINICAL STAGE**	
Stage I	20 (3.8%)
Stage II	98 (18.8%)
Stage III	106 (20.4%)
Stage VIA/VIB/VIC	282 (54.2%)

Not Available	14 (2.7%)
**TOTAL**	520 (100.0%)
